# North London Consumption Hospital

**Published:** 1899-10-07

**Authors:** 


### NORTH LONDON CONSUMPTION HOSPITAL.

The report of the result of the open-air treatment in consumption which emanates from the North London Hospital for Consumption, at Hampstead, is most encouraging, as showing that London patients need not go far afield to test the benefits of the method.

